# A case report of primary colonic paraganglioma with lymph node metastasis

**DOI:** 10.3389/fsurg.2022.961514

**Published:** 2022-08-09

**Authors:** Xinyi Zhu, Zhen Huang, Lin Dong, Hong Zhao, Haizhen Lu

**Affiliations:** ^1^ Department of Pathology, National Cancer Center/National Clinical Research Center for Cancer/Cancer Hospital, Chinese Academy of Medical Sciences and Peking Union Medical College, Beijing, China; ^2^ Department of Hepatobiliary surgery, National Cancer Center/National Clinical Research Center for Cancer/Cancer Hospital, Chinese Academy of Medical Sciences and Peking Union Medical College, Beijing, China

**Keywords:** paraganglioma, colon, lymph node metastasis, case report, NGS - next generation sequencing

## Abstract

**Background:**

Paraganglioma is a kind of neuroendocrine tumor that originates from paraganglia outside the adrenal gland. Gastrointestinal tract paraganglioma is very rare and only four cases of paraganglioma originating in the colon have been reported.

**Case Presentation:**

We report a case of metastatic paraganglioma originating in the colon, in which the differential diagnosis was established by comprehensively considering clinical information, histology, immunohistochemistry, and findings of fluorescence *in situ* hybridization and next generation sequencing analyses. The patient has remained well for over 14 months after the treatment.

**Conclusion:**

Since all paraganglioma have metastatic potential, we believe that radical surgical resection and regular follow-up are necessary. Genetic testing may be indicative of metastatic potential and prognosis. Because colonic paraganglioma is very rare, differential diagnosis is very important. Our report provides experience for the diagnosis and study of paraganglioma in rare sites.

## Introduction

Paraganglia originate from the neural crest and include sympathetic ganglia (such as adrenal medulla) and parasympathetic ganglia (such as the carotid body) (1). Both pheochromocytoma and paraganglioma (PPGL) originate in the paraganglia, and the two are distinguished by the anatomical location of the tumor. Pheochromocytoma occurs in the adrenal medulla, and paraganglioma occurs in the paraganglia other than the adrenal gland. The incidence of PPGL is approximately 0.6 cases per 100,000 people (2). The peak incidence of PPGL is observed in 20–40-year-old individuals, the average age of onset of hereditary cases is 24.9 years, the average age of onset of sporadic cases is 43.9 years, and there are no differences among the incidences in terms of sex (3). In the 2017 WHO classification of endocrine tumors, PPGLs were classified as malignant tumors from previous benign, borderline, or biologically uncertain tumors. The WHO believes that all PPGLs have metastatic potential, so PPGL is classified as metastatic and non-metastatic types. The diagnosis was added to the 8th edition of the TNM staging system of the American Joint Committee on Cancer (1, 4). PPGL is mostly sporadic and partly hereditary, which is related to a variety of gene mutations. Current studies have reported a number of molecular changes related to poor prognosis of PPGL. Therefore, in addition to the application of conventional auxiliary diagnosis methods, this case also completes the molecular examination, in order to provide ideas and supplements for the patient's follow-up diagnosis and treatment.

## Case presentation

### General information

A 49-year-old male patient presented to our hospital for examination with colonic space-occupying lesions that had been present for 8 months. The patient had elevated blood pressure before the operation, which was as high as 150/110 mmHg; however, he was unable to remember the time when the elevated blood pressure was identified. After consuming antihypertensive drugs, the name of which the patient was unable to recall, for 3 months, his blood pressure did not decrease. The patient sought medical intervention because of stool irregularities and a colonoscopy revealed ulcerative masses at 20–25 cm from the anal margin and multiple polyps in the rectum. Computed tomography showed irregular thickening and enhancement of the circumferential intestinal wall of the sigmoid colon, with a maximum diameter of approximately 5.9 cm. In addition, there were multiple lymph-node metastases in the mesentery, retroperitoneal, and left iliac vessels, with the largest being approximately 3.0 cm. Pathological analysis of a tumor biopsy specimen indicated that the tumor may be a paraganglioma. The patient underwent surgery for appendicitis and left leg trauma. The patient denied any history of radiation or chemical exposure. The patient had smoked for more than 20 years and drank a small amount of alcohol. All the family members are in good health.

### Treatment

The patient received preoperative neoadjuvant chemotherapy with tegafur and temozolomide orally for four cycles (specific dose: tegafur 60 mg orally twice a day; temozolomide 300 mg orally once a day). The maximum diameter of tumor and metastatic lymph nodes decreased after neoadjuvant chemotherapy (**Supplementary Figure S1**). The patient underwent sigmoid colon resection, omentectomy, abdominal wall nodule resection, and retroperitoneal lymph-node dissection on the sacrum.

### Pathological diagnosis

**Figure 1:**
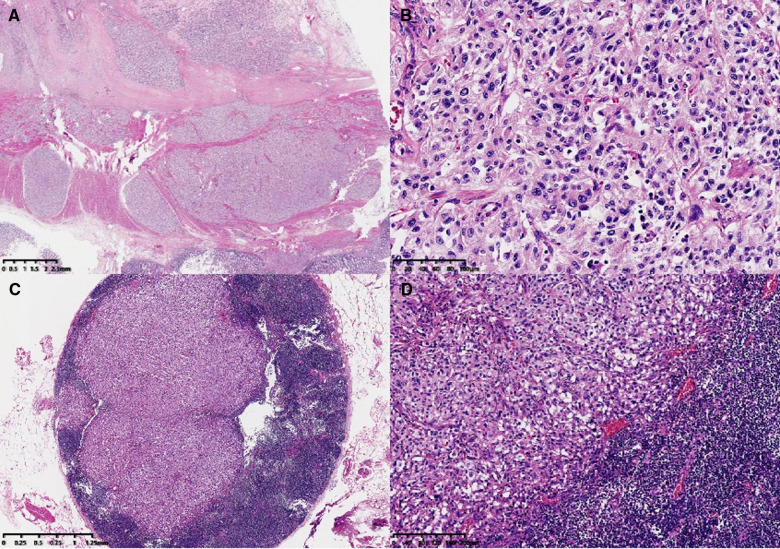
Microscopic features. The main body of the tumor was located in the muscularis and subserosa of the colonic wall (**A**, ×6). At high magnification, the tumor morphology appeared as a nest-like and organ-like structure (Zellballen pattern) (**B**, ×200). Lymph-node metastasis (**C**, ×20; **D**, ×100).

**Figure 2:**
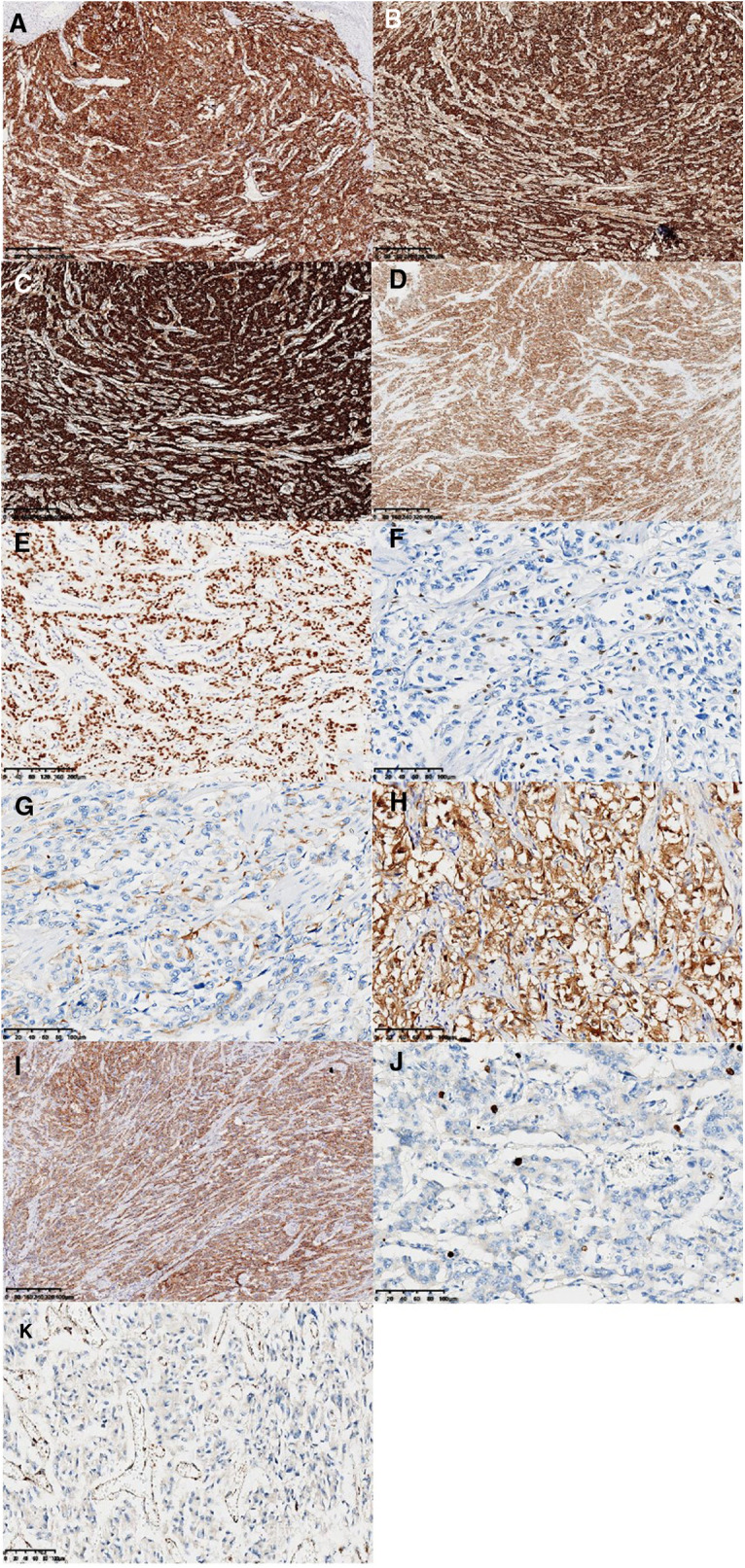
Immunohistochemical staining results. Neuroendocrine markers: CD56, ChrA, Syno, and NSE were diffusely and strongly positively expressed (**A**, ×40; **B**, ×40; **C**, ×40; **D**, ×40). GATA3 was diffuse nucleus positive (**E**, ×100). The sustentacular cells were highlighted by SOX10, GFAP, and S-100 immuno-stain (**F**, ×200; **G**, ×200; **H**, ×200). MelanA showed diffuse moderate expression (**I**, ×40). The Ki-67 labeling index was approximately 5% (**J**, ×200). SDHB showed negative expression (**K**, ×200).

The main body of the tumor was located in the muscularis and subserosa of the intestinal wall and had invaded the local serous membrane and lamina propria of the mucosa. Morphologically the tumor resembled a nest-like and organ-like structure. The tumor cells showed abundant cytoplasm, obvious nuclear atypia, and had a mitotic figure of <1 per 10 high-power fields. Mild lymphovascular invasion and nerve invasion were observed. Some tumor cells were slightly degenerative with interstitial hyalinosis, which was consistent with mild post-treatment reaction. In addition, metastatic tumors in lymph nodes (18/33) and tumor nodules could also be visualized (Figure. 1). Since no other tumors were seen during the patient's imaging examination and surgery, the primary tumor of the colon was first considered. Immunohistochemical staining results (Figure. 2): Epithelial markers, such as AE1/AE3, CK18, and EMA, were negative, indicating that the diagnosis of poorly differentiated adenocarcinoma and neuroendocrine tumors could be excluded. Neuroendocrine markers, such as CD56, Chromagranin-A, Synaptophysin, SSTR2, and NSE, were diffusely, strongly, and positively expressed, which supported the diagnosis of paraganglioma. Immunostaining for S-100, SOX10, and GFAP highlighted sustentacular cells, which can be found in varying numbers at the periphery of Zellballen or interspersed between tumor cells. HMB-45 was negative and MelanA showed diffuse moderate expression that cannot exclude the diagnosis of clear-cell sarcoma, so molecular testing is needed for further diagnosis. In addition, Desmin, SMA, CD117, and TTF-1 were all negative. Vimentin showed diffuse and strong cytoplasmic positive. GATA3 was diffuse nucleus positive, Inhibin was focal positive, and CD34 was vascular positive. The Ki-67 label index was approximately 5%. SDHB was diffusely negative, representing mutant expression. Reagent information can be found in **Supplementary Table 1**.

Differential diagnosis: Gastrointestinal clear-cell sarcoma, epithelial gastrointestinal stromal tumor, poorly differentiated adenocarcinoma, colonic neuroendocrine tumor and composite gangliocytoma/neuroma and neuroendocrine tumor.

Final pathological diagnosis: Colonic paraganglioma with lymph-node metastasis (18/33). According to GAPP score, the tumor was diagnosed as moderate differentiated type.

### Genetic mutation tests

*EWSR1* (22q12) chromosome translocation was negative, as shown through a fluorescence in-situ hybridization test. The result did not support a diagnosis of clear-cell sarcoma.

**Figure 3:**
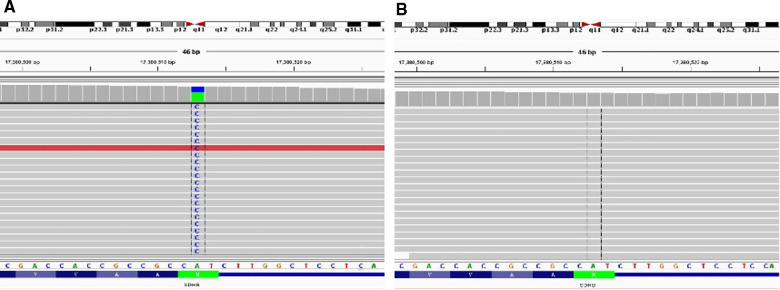
Comparison of tumor (**A**) and normal (**B**) FFPE samples. The *SDHB* gene somatic mutation (c.2 T > G, p.M1?) in exon 1 was detected by the NGS.

The DNA from paired tumor and normal formalin-fixed paraffin-embedded samples was extracted for next-generation sequencing (NGS). The *SDHB* gene somatic mutation (c.2 T > G, p.M1?) in exon 1 was detected by the NGS (Figure. 3). The mutation in exon 26 of the *ATRX* gene (p.R2028*) was also detected.

### Follow-up and outcome

The patient recovered well after the surgery and was actively followed-up. The patient’s blood pressure was normal and stable after surgery. Up to now, no recurrence or metastasis has been observed in 17 months after the operation.

## Discussion

The location of paraganglioma is basically the same as the normal distribution area of paraganglion in the human body. Paraganglioma is common in the carotid body, abdominal aorta, renal hilum, and inferior cavitary artery. Review of the domestic and foreign literature showed that paragangliomas that occur in the gastrointestinal tract are very rare and more common in the stomach, and there are a few literature reports of origination in the rectum (5). Colonic paragangliomas are extremely rare, with only 4 cases having been reported in the literature (6–9). The first case of colonic paraganglioma was reported by Yao et al. in 1997 (7). None of the four reported cases of colonic paraganglioma had metastases. In addition, owing to the earlier time, immature technology, or different professional focus, some cases of colonic paraganglioma that have been reported have limited clinical and pathological data. We reported our case of metastatic paraganglioma presenting as a primary colonic neoplasm with the goal of broadening the clinical and morphological spectrum and providing more complete and detailed clinical and pathological data, which will provide empirical support for any future study of paragangliomas in rare locations.

Analysis of five cases of colonic paraganglioma, including the present case, showed that the patients' male-to-female ratio was 2:3, and their respective ages were 12, 40, 49, 58, and 78 years (6–9). The average maximum diameter of the tumors was 3.16 cm. The main body of the tumor in the present case was located in the muscular layer and subserosal layer of the intestinal wall, and the tumors in the remaining most cases were mainly in the submucosa. These five cases of tumor cells were all arranged in nests with Zellballen pattern and abundant interstitial blood vessels. The immunohistochemical results were similar to those in our case; all cases were positive for neuroendocrine markers, and S-100 highlighted sustentacular cells. Furthermore, there are currently three reports of paraganglioma originating in the rectum, and we concluded that the microscopic morphological features and immunohistochemical results were similar to our case, with main organ-like nests (3, 5, 8). However, the cell proliferation activity in these three rectum-origin cases was high, and the index of Ki-67 was 20%, 41%, and 50%, respectively (3, 5, 8) Among the eight cases mentioned above, only three performed SDHB immunohistochemical staining and were diagnosed with SDHx-related paragangliomas. Only the case we reported performed next-generation sequencing.

Most PPGLs are sporadic, but studies have shown that approximately 40% are hereditary tumors and associated with germline and/or somatic mutations in ≥20 known susceptibility genes (10). Among these susceptibility genes, the most important and common ones are succinic dehydrogenase x (*SDHx*), receptor tyrosine kinase (*RET*), *VHL* (von Hippel-Lindau), and nerve neurofibromatosis type 1 (neurofibromatosis type 1, *NF1*), which come from a wide range of functional categories, and are related to the mechanism of tumorigenesis (10). Studies have found that the time to distant metastasis, local recurrence or regional lymph-node metastasis in patients with PPGL is significantly related to multiple molecular markers, including the *MAML3* fusion gene, *SDHB* germline mutation, somatic mutation of *SETD2* or *ATRX* gene, hypersomatic mutation, *Wnt* pathway changes, and hypermethylated subtypes. The above molecular changes will lead to a decrease in disease-free survival time; on the contrary, plasma and/or urine metanephrine or adrenaline positive, kinase signal expression, and hypomethylation are associated with longer disease-free survival time (11). Current studies have also found that changes in the *TERT*, *RDBP*, and *FH* genes are related to the poor prognosis of PPGL (12–14). In this case, NGS sequencing of tumor-related genes showed different degrees of variation in the *SDHB* gene and *ATRX* gene. Moreover, the lymph-node metastases in this case were relatively numerous, reaching 18/33, which also confirmed the results of the afore-mentioned study on the correlation between the *SDHB* gene and *ATRX* gene mutations and poor prognosis.

The definition of metastatic PPGL is the presence of metastatic lesions when recurrence or local tumor infiltration is excluded. The incidence of metastatic PPGL is <1 per 1,000,000. The metastasis of paraganglioma is more common than that of pheochromocytoma, and metastasis observed at the initial diagnosis is even rarer, occurring in approximately 10% of pheochromocytomas and approximately 34% of paragangliomas (15). The most common metastatic sites in patients with PPGL are lymph nodes, bone, liver, and lung (16). In this case, metastatic carcinoma was present in more than half of the lymph nodes. So far, recurrence or metastasis after surgery has not been observed in this patient, and he continues to take tegafur and temozolomide. The 5-year survival rate of patients with metastatic PPGL ranges from 12% to 84% (10). A meta-analysis of 1,338 patients with metastatic PPGL showed that the 5- and 10-year mortality rates were 24%–51% (seven studies, *n* = 738) and 17%–42% (two studies, *n* = 55) (17). A multi-center retrospective study of 169 patients with metastatic PPGL showed that a median survival time of 6.7 years, site of occurrence (neck and skull base), age <40 years, less than a five-fold increase in the level of catecholamines, and a low proliferation index (mitosis ≤3 per 10 high-power fields and/or Ki-67 ≤ 2%) were associated with better prognosis (18).

The current treatment options for metastatic PPGL include surgical resection, targeted radiolabeled carriers (such as 131I-MIBG or 90Y-DOTATE or 177LUTATE), thermal ablation, chemotherapy, and radiotherapy (2). So far, however, there is no standard chemotherapy regimen for the treatment of metastatic PPGL. Cyclophosphamide, vincristine, and dacarbazine (CVD) chemotherapy regimens are currently used to treat metastatic PPGL. A retrospective study showed that about one-third of patients experienced complete or partial remission after receiving CVD treatment, presenting tumor shrinkage and decreased secretion of catecholamines. This effect is maintained over time but not all patients with metastatic PPGL are suitable for CVD, and some patients have disease progression (10).

Colonic paraganglioma needs to be differentiated from the tumors described below.

(1) Clear-cell sarcoma of the gastrointestinal tract: This tumor is derived from the primitive melanocytes produced by the neural crest during the embryonic stage. Tumor cells are relatively uniform in size, mostly round, occasionally spindle shaped, and mostly arranged in nests or bundles separated by fibrous spacers. In this case, the expressions of S-100 and MelanA adversely affected differential diagnosis. In paraganglioma, S-100 is only found in the sustentacular cells around the cell nest. In clear-cell sarcoma, S-100 is more strongly expressed and may be accompanied by HMB-45 and MelanA positivity. Molecular testing can also help differential diagnosis of gastrointestinal clear-cell sarcoma; its characteristic EWSR1-ATF1/ t(12; 22) (q13; q12) chromosome translocation can be used as a basis for molecular pathological diagnosis.
(2) Colon adenocarcinoma: The immunophenotype of colonic paraganglioma has certain characteristics. The tumor cell expresses neuroendocrine markers, such as Syno, ChrA, CD56, and NSE, but not the epithelial markers CK and EMA. The above characteristics can be distinguished from colon adenocarcinoma.
(3) Colonic neuroendocrine tumors (NETs): Although both NETs and paraganglioma express neuroendocrine markers, NETs express epithelial markers, whereas paraganglioma do not. In addition, GATA3 is reported to be negative in NETs and positive in paraganglioma which also helps to distinguish (19).
(4) Epithelial gastrointestinal stromal tumors: Gastrointestinal stromal tumors are derived from Caja cells in the intestinal wall and specifically express the molecular markers DOG-1 and CD117, which can be used for differential diagnosis.
(5) Composite gangliocytoma/neuroma and neuroendocrine tumor (CoGNET): Paraganglioma lacks ganglion cells and expresses GATA3. CoGNET commonly occurs in the duodenum, and almost always express cytokeratins. But CoGNET is negative for GATA3 (4). In addition, molecular alterations in paraganglioma such as SDHx mutations can also help in the differential diagnosis.

Colonic paraganglioma is a very rare primary colon tumor. Its prognosis may be related to a variety of molecular changes. A clear diagnosis should be based on histopathological morphology, immunophenotype, and molecular diagnosis to identify other tumors that may occur in the colon. And surgery should be performed as soon as possible.

## Data Availability

The datasets presented in this article are not readily available because The data are not publicly available due to hospital regulations. But data requests with aims will be needed to assess the reasonability. After approval from the hospital and the corresponding authors, de-identified clinical data will be provided. Requests to access the datasets should be directed to Haizhen Lu, luhz@cicams.ac.cn.
