# Supplementary material for: A case report of primary colonic paraganglioma with lymph node metastasis
Source: Front Surg. 2022 Aug 9;9:961514. doi: 10.3389/fsurg.2022.961514 (PMC9395918; doi:10.3389/fsurg.2022.961514)
Supplement: Supplementary file 1 [file Table_1_v1.docx]

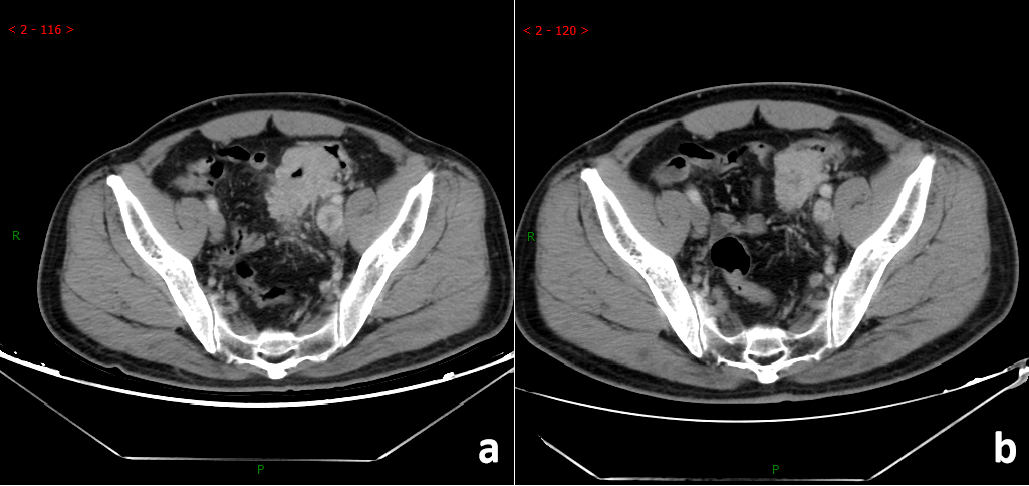


**Supplementary Figure 1:** Comparing the computed tomography image before neoadjuvant chemotherapy (a), the maximum diameter of tumor (red arrows) and metastatic lymph node (blue arrows) decreased after neoadjuvant chemotherapy (b).

**Supplementary Table 1: Materials used in experiments**

| IHC | | | |
| --- | --- | --- | --- |
| CK | Fuzhou Maixin Biotech, China, monoclonal AE1/AE3 | CD117 | Fuzhou Maixin Biotech, China, monoclonal YR145 |
| Vimentin | Beijing Zhong Shan-Golden Bridge Biotech, China, monoclonal UMAB159 | CD34 | Fuzhou Maixin Biotech, China, monoclonal QBEnd/10 |
| CD56 | Fuzhou Maixin Biotech, China, monoclonal MX039 | CK18 | Fuzhou Maixin Biotech, China, monoclonal MX035 |
| ChrA | Fuzhou Maixin Biotech, China, monoclonal LK2H10+PHE5 | EMA | Fuzhou Maixin Biotech, China, monoclonal E29 |
| Syno | Fuzhou Maixin Biotech, China, monoclonal SP11 | TTF-1 | Ventana, America, monoclonal SP141 |
| SSTR2 | Beijing Zhong Shan-Golden Bridge Biotech, China, monoclonal EP149 | HMB45 | Fuzhou Maixin Biotech, China, monoclonal HMB45 |
| S-100 | Fuzhou Maixin Biotech, China, monoclonal 4C4.9 | Desmin | Fuzhou Maixin Biotech, China, monoclonal MX046 |
| Inhibin | Beijing Zhong Shan-Golden Bridge Biotech, China, monoclonal AMY82 | SMA | Fuzhou Maixin Biotech, China, monoclonal 1A4 |
| Melan-A | Beijing Zhong Shan-Golden Bridge Biotech, China, monoclonal A103 | Ki-67 | Beijing Zhong Shan-Golden Bridge Biotech, China, monoclonal MIB1 |
| GFAP | Fuzhou Maixin Biotech, China, monoclonal GA-5/6F2 | GATA3 | Beijing Zhong Shan-Golden Bridge Biotech, China, monoclonal EP368 |
| NSE | Fuzhou Maixin Biotech, China, monoclonal 3-3-C | SOX10 | Beijing Zhong Shan-Golden Bridge Biotech, China, monoclonal EP268 |
| SDHB | Beijing Zhong Shan-Golden Bridge Biotech, China, monoclonal OTI1H6 |  |  |
| FISH: Vysis LSI, America, EWSR1 Break Apart FISH Probe Kit | | | |
| NGS: Burning Rock Biotech, China, OncoScreen Plus Cancer Mutation Profiling Tissue Kit | | | |
